# A Transfer Learning Approach for Clinical Detection Support of Monkeypox Skin Lesions

**DOI:** 10.3390/diagnostics13081503

**Published:** 2023-04-21

**Authors:** Maram Fahaad Almufareh, Samabia Tehsin, Mamoona Humayun, Sumaira Kausar

**Affiliations:** 1 Department of Information Systems, College of Computer and Information Sciences, Jouf University, Sakakah 72388, Saudi Arabia; mfalmufareh@ju.edu.sa; 2 Department of Computer Science, Bahria University, Islamabad 44220, Pakistan; samabiatehsin.bukc@bahria.edu.com (S.T.);

**Keywords:** monkeypox, transfer learning, computer-aided diagnosis, skin lesion detection, artificial intelligence, deep learning, IoT

## Abstract

Monkeypox (MPX) is a disease caused by monkeypox virus (MPXV). It is a contagious disease and has associated symptoms of skin lesions, rashes, fever, and respiratory distress lymph swelling along with numerous neurological distresses. This can be a deadly disease, and the latest outbreak of it has shown its spread to Europe, Australia, the United States, and Africa. Typically, diagnosis of MPX is performed through PCR, by taking a sample of the skin lesion. This procedure is risky for medical staff, as during sample collection, transmission and testing, they can be exposed to MPXV, and this infectious disease can be transferred to medical staff. In the current era, cutting-edge technologies such as IoT and artificial intelligence (AI) have made the diagnostics process smart and secure. IoT devices such as wearables and sensors permit seamless data collection while AI techniques utilize the data in disease diagnosis. Keeping in view the importance of these cutting-edge technologies, this paper presents a non-invasive, non-contact, computer-vision-based method for diagnosis of MPX by analyzing skin lesion images that are more smart and secure compared to traditional methods of diagnosis. The proposed methodology employs deep learning techniques to classify skin lesions as MPXV positive or not. Two datasets, the Kaggle Monkeypox Skin Lesion Dataset (MSLD) and the Monkeypox Skin Image Dataset (MSID), are used for evaluating the proposed methodology. The results on multiple deep learning models were evaluated using sensitivity, specificity and balanced accuracy. The proposed method has yielded highly promising results, demonstrating its potential for wide-scale deployment in detecting monkeypox. This smart and cost-effective solution can be effectively utilized in underprivileged areas where laboratory infrastructure may be lacking.

## 1. Introduction

The use of AI (artificial intelligence) in the diagnosis of diseases has great potential to improve healthcare outcomes. AI refers to the ability of machines to learn and perform tasks that typically require human intelligence [1]. The Internet of Things (IoT) is a concept that refers to the connectivity of devices and objects through the internet, allowing them to collect and transmit data in real time. In healthcare, IoT has enormous potential, particularly in the field of medical imaging [2]. Medical imaging techniques generate vast amounts of data in the form of images, which can be challenging for human experts to interpret accurately. These images may contain subtle patterns or abnormalities that require specialized expertise to detect and analyze, and misinterpretation can lead to inaccurate diagnoses and treatment plans. AI can help solve this problem. AI algorithms can be trained on large datasets of medical images, utilizing techniques such as deep learning, machine learning, and computer vision to identify patterns, anomalies, and features that may be indicative of diseases [3,4]. These algorithms can process and analyze medical images in real time, aiding healthcare providers in making more accurate and timely diagnoses.

By combining IoT and AI in medical imaging, healthcare professionals can access sophisticated tools that can enhance their diagnostic capabilities [5]. For example, AI algorithms can automatically detect early signs of diseases such as cancer, cardiovascular conditions, and neurological disorders from medical images with high accuracy, even in cases where the abnormalities are subtle and are not easily discernible by human experts. Moreover, IoT-enabled medical imaging devices can transmit image data securely to remote locations, allowing for telemedicine and telediagnosis, particularly in underserved areas where access to specialized medical expertise may be limited. The combination of IoT and AI in medical imaging has the potential to revolutionize disease diagnosis and patient care by improving the accuracy, efficiency, and accessibility of diagnostic processes. It enables healthcare providers to harness the power of data and advanced analytics to make more informed decisions, leading to better patient outcomes and ultimately improving the quality of healthcare services [6].

Viruses are infectious agents that can enter the human body and cause diseases, malfunction or dysfunction of human organs. Monkeypox virus is a DNA virus that was first observed in humans in 1970 [7]. MPXV causes an illness, named as monkeypox. Monkeypox has typical symptoms such as rash of numerous lesions in multiple areas of the body, typically on the face, arms, legs, trunk and sometimes on palms and soles [8,9]. Apart from rashy lesions, MPX also has symptoms such as fever, respiratory distress and lymphadenopathy [10,11,12]. MPX can be fatal. It poses high risks of mortality for people with existing medical conditions and weak immune systems. It can also cause neurological manifestations including headache, fatigue, muscle pain, loss of appetite, and changes in mental alertness [13,14]. MPX typically lasts from 2–4 weeks with variant levels of severity. It is a contagious disease that can spread through skin–skin contact and exposure to respiratory secretions. It can be spread through close contact with someone who is infected by touching the rash area or bodily fluids, or by indirect contact such as touching objects that are used by the MPX infected. MPX is normally considered as endemic, as it is mostly observed in some areas of Africa, but the recent outbreak in mid-2022 has shown its spread to many other regions of Africa and a large area of Europe along with the United States, Canada and Australia [15,16,17].

The MPX diagnostic test is performed on lesion samples as per the guidelines from the US Food and Drug Administration (FDA). A swab is rubbed on the lesion to take a sample, or alternatively the lesion crust can be taken as the sample. Then, this sample is placed in some VTM (viral transport media) and is transferred to the lab for examination of MPXV using polymerase chain reaction (PCR) systems [18].

As it was mentioned that MPX is contagious and the latest outbreak is quite alarming, there is a dire need to improve the diagnosis process. The prevailing method of testing requires personal exposure to the MPXV, as there is a high chance of transmission of the virus while taking, transmitting and testing the lesion samples for diagnosis. This paper presents an effective method for diagnosis of MPX using computer vision techniques. This computer-vision-based diagnosis of monkeypox can avoid the required contact and exposure of medical staff to MPXV while testing.

### 1.1. Motivation and Contribution

Automated systems for monkeypox detection can provide several benefits compared to traditional diagnostic methods. One of the main motivations for developing such a system is to improve the accuracy and speed of diagnosis.

Monkeypox is a viral disease that can cause a range of symptoms, including fever, rash, and respiratory issues. These symptoms can be similar to those of other viral diseases, such as smallpox and chickenpox, making diagnosis challenging for healthcare professionals. An automated system that uses AI and machine learning algorithms can analyze large amounts of data to identify specific patterns and markers that are unique to monkeypox, thus providing a more accurate diagnosis. Furthermore, an automated system can reduce the time it takes to diagnose monkeypox, allowing for quicker treatment and isolation of patients. This is particularly important in outbreak situations, where rapid detection and response can help contain the spread of the disease.

This research on monkeypox detection using transfer learning makes several contributions to the field of biomedical image analysis and disease diagnosis. Below is a compilation of the various contributions made:

- Improved accuracy of monkeypox detection: By using transfer learning, we have achieved the improved accuracy of monkeypox detection.
- Better understanding of the features that distinguish monkeypox: By analyzing the features and patterns learned by the deep learning model, we present insights into the characteristics that distinguish monkeypox from other diseases or healthy tissue. This can help to improve our understanding of the disease and inform future research.
- Improved disease surveillance: Monkeypox is a rare disease, and detecting it early is crucial for preventing its spread. By developing a more accurate and efficient method for detecting monkeypox using transfer learning, researchers have contributed to better disease surveillance and control.

### 1.2. Paper Organization

The introduction section is followed by a review of related work, where the current state of the art in the field is discussed, and the gap in knowledge that the current research aims to address is identified.

The methodology section is presented next, which details the proposed algorithms and techniques used to collect, preprocess, analyze, and interpret the data, feature extraction and classification. This section also describes the model architecture used. The results and analysis section presents the findings of the study, including the evaluation metrics, hyperparameters, training, and validation procedures used to assess the performance of the model, and the statistical analysis of the results. It also includes the analysis where the results are interpreted in the context of the research problem, and the implications of the findings are discussed. Finally, the paper concludes with a summary of the main contributions and the potential impact of the study. References are included at the end of the paper, which list the sources cited in the text.

## 2. Related Work

Skin lesions are a common occurrence in clinical practice, and it is crucial to accurately detect and diagnose them for proper patient management. In recent years, the emergence of artificial intelligence (AI) and deep learning techniques has shown great potential in aiding clinical decision making for skin lesion recognition [19,20,21]. This literature review provides an overview of relevant studies related to skin problems, including various skin lesions and conditions, as well as studies specifically focused on detecting monkeypox skin lesions. The first section summarizes the existing literature on AI-based approaches for detecting and diagnosing skin problems, such as transfer learning, convolutional neural networks (CNNs), and other machine learning algorithms. The second section focuses on studies related to monkeypox detection, including the use of different datasets, image preprocessing techniques, and model architectures. The aim of this review is to provide a comprehensive understanding of the current state of the field and the potential of AI-based approaches for supporting clinical detection of monkeypox skin lesions.

### 2.1. Skin Lesion Detection and Diagnosis Using AI

Melanoma is a form of skin cancer that starts in the cells that produce pigment in the skin, known as melanocytes. These cells produce the pigment melanin, which provides color to the skin, hair, and eyes. Melanoma can be found in many places in the body; however, it is mainly seen in areas that are exposed to the sun such as the face, neck, arms, and legs, as well as in areas that are not exposed to the sun, such as the palms of the hands and soles of the feet. Deep-learning-based methods are proposed by [22,23] for the diagnosis of melanoma using visual cues. Additionally, Yadav et al. implemented deep learning for facial skin disease detection [24].

Sandeep et al. [25] studied the application of deep learning (DL) for spotting numerous skin conditions, including chickenpox, vitiligo, psoriasis, acne, melanoma, lupus, ringworm and herpes. They constructed a convolutional neural network (CNN) to categorize skin lesions into eight disease classes. An accuracy of 78% for the detection was achieved by their model. Glock et al. [26] proposed a transfer learning approach for measles detection, leveraging the ResNet-50 model. This approach demonstrated a sensitivity of 81.7%, specificity of 97.1%, and accuracy of 95.2% on a varied rash image dataset.

### 2.2. Monkeypox Detection Using AI and Deep Learning Techniques

Ali, Shams Nafisa et al. [19] presented the open-source “Monkeypox Skin Lesion Dataset (MSLD)” to enable automated detection of the Monkeypox disease from skin lesions. Deep learning methods have proven to be effective for this purpose, provided that enough training examples are available. However, prior to this initiative, such a dataset was not available. A transfer learning approach using CNN architectures, namely VGG16 [27], ResNet50 [28], and InceptionV3 [29], was used. They were pretrained on ImageNet dataset. The performance of the three pretrained models (ResNet50, VGG16 and an ensemble system) was assessed via a three-fold cross-validation experiment. The results showed that ResNet50 achieved the highest accuracy, followed by VGG16. The ensemble system was formed by majority voting, but it did not outperform the best-performing ResNet50 model. However, the ensemble had the lowest standard deviation of the accuracy metric, suggesting that its performance is more consistent across the three folds. Despite the small dataset, these promising results demonstrate the potential of using AI-assisted early diagnosis of the disease.

Thirteen different pretrained deep learning models were investigated and compared by Sitaula et al. [30] for the diagnosis of MPX using the dataset of Ahsan et al. [31]. The results were then analyzed using four measures, namely accuracy, recall, precision and F1-score. The best performing DL models were first identified. After identification, the models were then ensembled by majority voting to improve the overall performance. The three-fold cross-validation experiment evaluated the performance of the selected pretrained models. The ensemble approach was reported to have the highest results for monkeypox virus detection, with a Precision of 85.44%, a recall of 85.47%, an F1-score of 85.40%, and an accuracy of 87.13%. The researcher [32] proposed image classification to differentiate between monkeypox and measles, utilizing deep learning and the convolutional neural network (CNN) architecture in combination with VGG-16 transfer learning. This study employed the dataset of Kaggle by Bala et al. [33]. This [34] study combined multiple datasets to classify monkeypox, such as against chickenpox, measles, normal and all diseases. Majority voting achieved 0.97 accuracy for monkeypox vs. chickenpox, Xception 0.79 for monkeypox vs. measles, MobileNetV2 0.96 for monkeypox vs. normal, and Lenet 0.80 for monkeypox vs. all. These results were obtained by employing multiple CNN-based pretrained models and majority voting. The study in [35] examines the use of deep transfer learning combined with a convolutional block attention module (CBAM) to perform image-based classification of human monkeypox disease. The CBAM is intended to focus on the most relevant parts of the feature maps for accurate classification. Ref. [36] proposed a transfer learning model for monkeypox detection using the MSLD dataset.

Table 1 shows the summary of related work in the diagnosis of monkeypox. Limited research exists on employing machine learning for diagnosing monkeypox. A few investigations have examined the capability of machine learning algorithms in recognizing the sickness. These studies suggest that machine learning could be a beneficial tool for diagnosing and stopping monkeypox, yet further research is necessary to confirm these outcomes and to create useful applications for clinical settings.

## 3. Proposed Methodology

**Figure 1:**
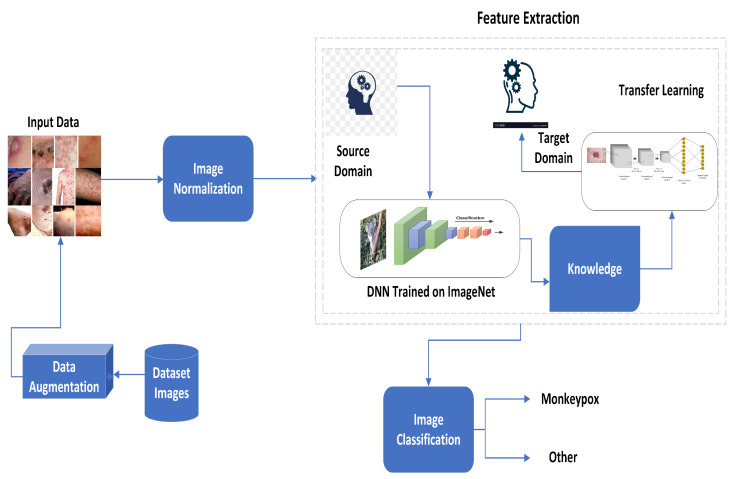
Architectural diagram of proposed method.

A fully automated, noninvasive deep learning approach is applied for detection of the presence of the virus. The proposed methodology takes the normal skin image as input and investigates for Monkeypox skin lesion. The proposed methodology first applies the pre-processing steps, then a feature map is extracted through transfer learning, and finally the input is classififed as monkeypox or other type of skin lesion. The complete architectural diagram is presented in Figure 1.

### 3.1. Data Acquisition and Preparation

Two datasets are used for the experimentation of this research, namely, the Kaggle Monkeypox Skin Lesion Dataset (MSLD) and the Monkeypox Skin Image Dataset (MSID). Table 2 shows the details of the datasets. The MSID dataset comprises four distinct categories: monkeypox, chickenpox, measles, and normal, but all are merged as the others class for uniformity of the problem. These image classes were sourced from various online platforms. The Department of Computer Science and Engineering at Islamic University in Kushtia-7003, Bangladesh, was responsible for creating the entire dataset. The MSLD is generated through the collection and analysis of images obtained from various sources of web-scraping, such as news portals, websites, and publicly available case reports. The main objective of creating the MSLD is to differentiate between monkeypox cases and other similar non-monkeypox cases. To achieve this, the MSLD includes skin lesion images of chickenpox and measles, which bear a resemblance to the rash and pustules of monkeypox in its initial stages. These images are classified into two categories: “monkeypox” class and “others” class, which perform binary classification.

**Figure 2:**
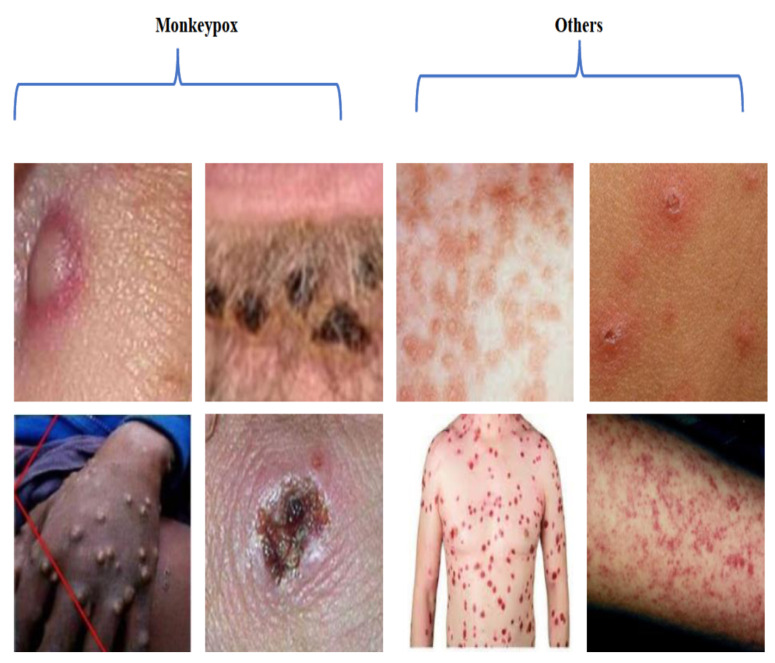
Sample images of datasets: first row shows images of MSLD and the second row shows MSID samples.

Figure 2 illustrates the sample images of both datasets.

**Figure 3:**
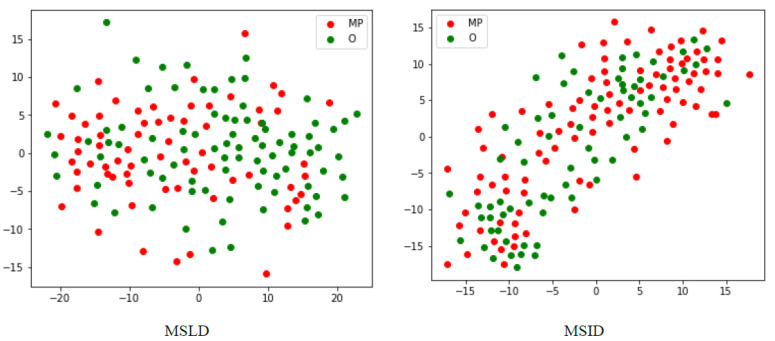
Dataset visualization for monkeypox and other class distribution.

Figure 3 shows the dataset visualization using the t-distributed stochastic neighbor embedding technique. The visualization shows the difficulty of the addressed problem. The inter-class variability is low while the intra-class variability is quite high.

To address the issue of scarcity of medical data, augmentation is applied. The data are augmented by applying the transformation function *ℶ* to the input image χ(ℵ(i,j)). The total of eight transformations is applied.

Therefore, $ℶ\;\in R_{\theta },\;{HS}_{s},\;\;{VS}_{t,},\;{Sh}_{u},\;Z_{v},\;{HF}_{w},\;{VF}_{x},\;B_{y}$.

(1) $$R_{\theta }\left(ℵ\left(i,j\right)\right)=ℵ\left(i*\cos\left(\theta \right)-j*\sin\left(\theta \right),i*\sin\left(\theta \right)+j*\cos\left(\theta \right)\right)$$

(2) $${HS}_{s}\left(ℵ\left(i,j\right)\right)=\;ℵ\left(i+s,j\right)$$

(3) $${VS}_{t}\left(ℵ\left(i,j\right)\right)=\;ℵ\left(i,j+t\right)$$

(4) $${Sh}_{u}\left(ℵ\left(i,j\right)\right)=\;ℵ\left(i+u*j,j\right)$$

(5) $$Z_{v}\left(ℵ\left(i,j\right)\right)=\;ℵ\left(i*v,j*v\right)$$

(6) $${HF}_{w}\left(ℵ\left(i,j\right)\right)=ℵ\left(M-i-1,j\right)\;$$

(7) $${VF}_{x}\left(ℵ\left(i,j\right)\right)=ℵ\left(i,N-j-1\right)\;$$

(8) $$B_{y}\left(ℵ\left(i,j\right)\right)=\;y*ℵ\left(i,j\right)$$

### 3.2. Preprocessing

The input ℵ(i,j) is a three-channel image piece of data with a spatial dimension as MxN. This means that the image *ℵ* has *M* rows and *N* columns, abd (i,j) points to the pixel location in the image. Pixel normalization is applied before heading toward the feature extraction module. The mapping function χ is applied on the input image. Mapping function χ:[0,255]→[0,1] is defined as

(9) $$\chi \left(ℵ{\left(i,j\right)}^{c}\right)=\frac{ℵ(i,j)}{max_{s}{,}_{t}ℵ\left(s,t\right)}$$

i∈[1,M],j∈[1,M] and C∈R,G,B.

### 3.3. Feature Extraction

Transfer learning is employed for feature extraction to detect monkeypox. Consider a source domain $\xi_{s}$ and target domain $\xi_{T}$. Let L(S) is the learning system of the source domain, obtained by mapping of the input–output training pairs $\left\{I_{1}^{S},\;I_{2}^{,S},\;\ldots \ldots ..\;I_{n}^{S}\right\}\epsilon R$. Then, for the target domain, the learning system L(S) adopts the knowledge of the source domain $\xi_{s}$ for the mapping of inputs and labels of the target problem domain. Hence, the L(T) utilizes this knowledge for pair mapping of $\left\{I_{1}^{T},\;I_{2}^{T},\;\ldots \ldots ..\;I_{m}^{T}\right\}\epsilon R$. This is adopted when m≪n. Transfer learning is utilized in medical diagnostic problems, because of a lack of annotated data. In this research, the $I_{i}^{S}$ is the imageNet sample pair, and $I_{j}^{T}$ is the sample of the monkeypox dataset.

After adopting the feature map $F^{S}$ from L(S), it is fed into the fully connected layer of one hundred units.

(10) $$℘=\varpi (\sum_{a=1}^{l}w_{a}1F_{a}^{S}+b_{a})$$

Here, *l* is the length of $F^{S}$, and ϖ is the activation function.

### 3.4. Image Classification

Image classification is a task in computer vision where an algorithm predicts the class or category of an input image. After extraction of the feature map through transfer learning, the classification results are obtained by a sigmoid activation function. The classification layer can be defined as

(11) $$ℷ\left(℘\right)=\;\frac{1}{1+\;e^{-℘}}\;$$

Weight regularization is used for avoiding overfitting. It penalizes those weights that become very large. Let $\ell \left(y,\hat{y}\right)$ denote the loss function, then after applying the regularization *g*, it will be defined as

(12) $$\ell^{\prime }\left(y,\hat{y}\right)=\;\ell \left(y,\hat{y}\right)+\left\|g\right\|\varphi$$

(13) $$g_{\ell_{1}}=\;\sum_{i=1}^{N}{g_{i}}^{2}$$

(14) $$g_{\ell_{2}}=\;\sum_{i=1}^{N}\left|g_{i}\right|$$

This research makes use of both $g_{\ell_{1}}$ and $g_{\ell_{2}}$ regularizations. The binary categorical cross entropy loss function is used in this research and can be defined as

(15) $$\ell \left(Out,\hat{Out}\right)=\frac{1}{size_{o}utput}\sum_{i=1}^{size_{o}utput}Out_{i}.\log{\hat{Out}}_{i}+(1-\;Out_{i}).\log{(1-\hat{Out}}_{i})$$

The early stopping rule is also employed for limiting the effect of a small dataset and overfitting. Algorithm 1 shows the complete algorithm for the proposed methodology.   
**Algorithm 1**: Algorithm for MonkeyPox Detection.
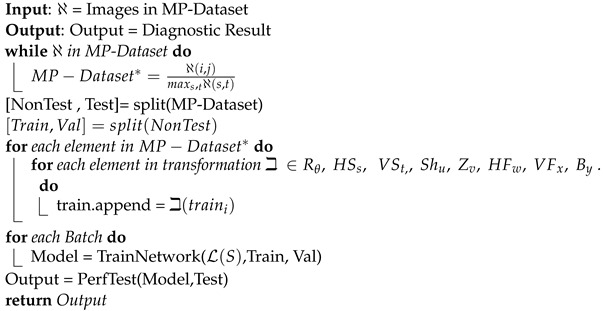


## 4. Results and Analysis

This part of the document presents the findings of the study and their interpretation. In this section, the data collected through the research process are analyzed, and the outcomes of the study are presented. It also provides a comprehensive overview of the research outcomes. The analysis of the data involves the use of various statistical techniques and visualization tools to draw meaningful insights from the collected data. In this section, we present the results and analysis section of our study, which investigates the effectiveness of the proposed methodology for the detection of MPX disease.

### 4.1. Experimental Setup

Transfer learning experiments involve several hyperparameters that need to be tuned to achieve optimal performance. These hyperparameters include learning rate, batch size, number of epochs, and regularization strength. A random search is used to explore the hyperparameter space and to find the optimal combination of values. Adam (short for adaptive moment estimation) is a popular optimization algorithm used for training the networks. It is an adaptive learning rate method that combines the advantages of two other popular optimization methods, Adagrad and RMSProp. Like Adagrad, Adam adapts the learning rate for each parameter based on the historical estimates of the gradient. This means that the learning rate is reduced for parameters that are updated frequently and is increased for parameters that are updated infrequently. In addition to the adaptive learning rate, Adam also uses momentum to accelerate the convergence of the optimization. Specifically, Adam computes a moving average of the gradient and its square, which serves as an estimate of the first and second moments of the gradient. These moments are then used to adjust the learning rate and the direction of the optimization, respectively. Early stopping is used to manage the number of epochs for training. Early stopping is a technique used in machine learning to prevent overfitting and to improve the generalization performance of a model. Monitoring the performance of the model on a validation set while training, and stopping the training when the performance begins to decline, is essential. It prevents overfitting, saves computational resources and improves generalization performance. All experiments were conducted on NVIDIA GeForce GTX 1060 GPU. Mini-batch was fixed for 16 for all the experiments.

### 4.2. Evaluation Metrics

We adopted four deep neural networks for transfer learning, namely, Inception V3, ResNet 50 V2, MobileNet V2 and EfficientNet- B4. The results were evaluated using three important metrics: sensitivity (ς), specificity (ϱ) and balanced accuracy (α). Besides these quantitative measures, ROC curves are also presented for visual analysis.

Sensitivity (ς), specificity (ϱ) and balanced accuracy (α) can be defined as

(16) $$\varsigma =\;\frac{TP}{\left(TP+FN\right)}$$

(17) $$\varrho =\frac{TN}{FP+TN}$$

(18) $$\alpha =BalancedAccuracy=\frac{\varsigma +\varrho }{2}$$

Here,

True positives (TP): The target and predicted output are both monkeypox.
True negatives (TN): The target and predicted output are both other.
False positives (FP): The target is other and the predicted output is monkeypox.
False negatives (FN): The target is monkeypox and the predicted output is other.

### 4.3. Experimental Results

Table 3 and Table 4 shows the comparative results of different architectures on MSID and MSLD datasets. MobileNet and Inception Nets perform better for both the datasets. MobileNet outperforms the other network on the MSID dataset with 96.55% balanced accuracy, 0.93 specificity and maximum sensitivity. For MSLD dataset, Inception V3 metrics proved best with 94% balanced accuracy, max specificity and 0.88 sensitivity. These two networks have simpler architecture as compared to ResNet and EfficientNet. Simple architectures perform better for small datasets. When the model is too complex, it can begin to learn the extraneous knowledge within the dataset. This results in degraded performance on unseen data.

**Figure 4:**
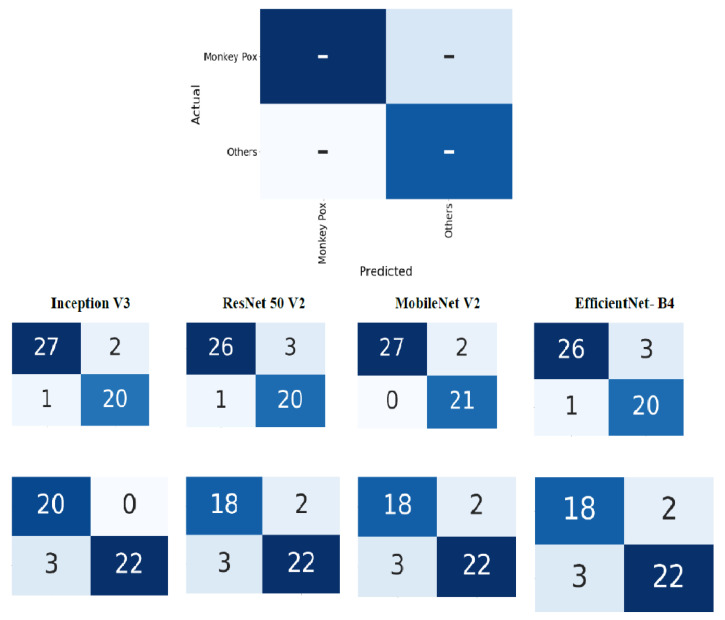
Confusion matrix for different architectures. Top row shows a sample of the labeled confusion matrix. THe middle row shows the results for MSID, and the bottom row shows them for MSLD.

Figure 4 shows the confusion matrix of all the architectures tested on the mentioned datasets. A confusion matrix is used for the evaluation of classification problems. The size of the matrix depends upon the output dimension of the classification problem. For binary classification, it is of 2 × 2 size. The matrix compares the target and predicted outputs for the classification model. Generating the confusion matrices allowed for a better comprehension of the results. After evaluation of the model, a confusion matrix showcased the genuine positives and negatives. This provided us with a clear understanding of any inaccurate predictions from the model, along with the number of genuine negatives or false positives. The utilization of the confusion matrix enabled us to conclude that the majority of predictions generated by the model were accurate. Figure 4 showcases the confusion matrix for the different architectures. Despite this, many of the images were too similar, leading to potential inaccuracies.

**Figure 5:**
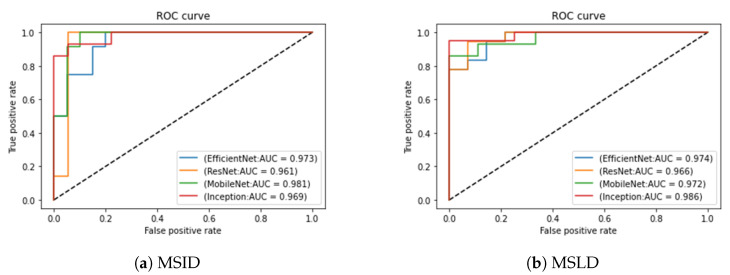
Comparison of ROC curve for different architectures.

Figure 5 presents the ROC (receiver operating characteristic) curves and corresponding AUC (area under the curve) values. ROC curves are a widely used metric in machine learning, especially in binary classification problems. They display the true positive rate (TPR) against the false positive rate (FPR) for various classification thresholds. TPR is the proportion of actual positive cases (true positives) identified correctly by the classifier, while FPR is the proportion of actual negative cases (true negatives) incorrectly classified as positive by the classifier. An ROC curve is a graphical representation of model performance on different thresholds. The higher the value of AUC, the better the performance of the model. A graphical depiction of the balance between the true positive rate and the false positive rate for a certain classifier is shown by the ROC curve, which helps in choosing the best threshold that balances the two rates according to the specific needs. The area under the receiver operating characteristic (ROC) curve (AUC) offers a single metric to assess the overall performance of a classifier, with a value of 1 indicating perfect classification and 0.5 indicating random guessing. For MSID data, inception Net gives the maximum AUC value, and for MSLD data, mobilenet performs best in terms of AUC.

**Figure 6:**
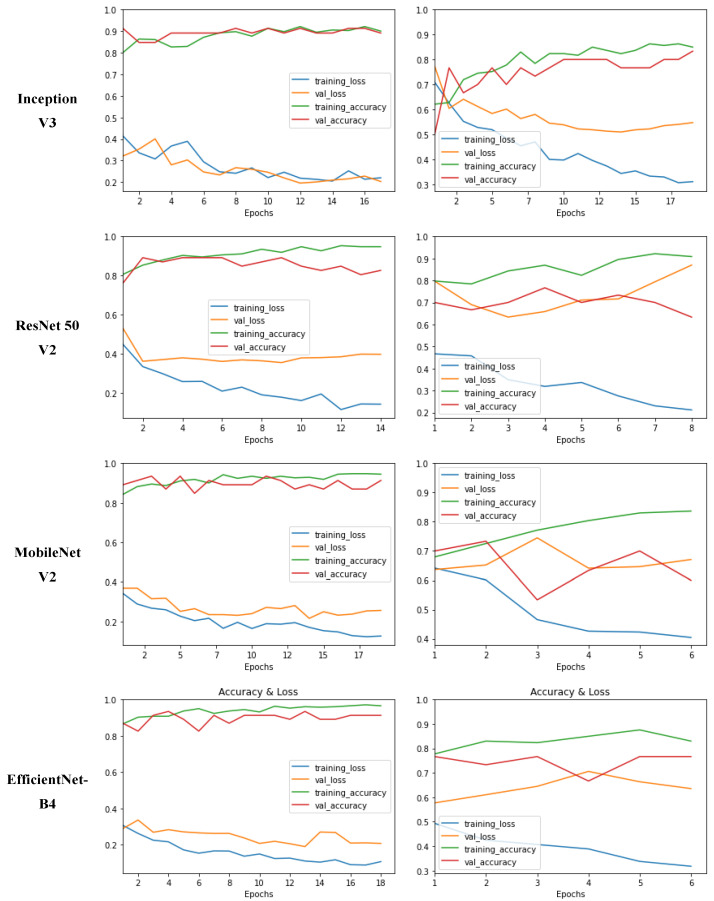
Accuracy loss curves for training and validation data. The first column shows the results for MSID and the second column shows them for MSLD.

Figure 6 shows the learning curves for all architectures. A learning curve is a plot of model performance (such as accuracy or loss) as a function of the amount of training data or number of epochs. The variations in epoch length are due to the early stopping feature. The graphs indicate that the accuracy of the model increases exponentially after the second epoch and that the validation accuracy was slightly lower than the training accuracy in each epoch. Additionally, the loss decreased in proportion to the number of epochs, reaching its lowest point at the end of training, indicating that the model was adequately trained and that the classification of monkeypox disease could be performed.

Table 5 is a comparison table that evaluates the performance of different methodologies on the MSLD dataset using different evaluation metrics. The table shows the performance of each methodology in terms of accuracy, specificity, sensitivity, and F1-measure. Table 5 compares the results of four methodologies by Nafisa et al. [19], Haque et al. [35], Sahin et al. [36] and the proposed one in this paper. The current proposed methodology has the highest accuracy of 0.93, which indicates that it correctly identifies 93% of the samples. The proposed methodology also has the highest F1-measure of 0.94, indicating that it has a balanced trade-off between precision and recall. Additionally, the proposed methodology has a specificity of 1, which means that it correctly identifies all negative samples.

The results of the other three methodologies are also shown in the table. Nafisa et al. [19] achieved an accuracy of 0.79, Haque et al. [35] achieved an accuracy of 0.83, and Sahin et al. [36] achieved an accuracy of 0.91. These values are lower than the proposed methodology’s accuracy. However, Sahin et al. [36] achieved the highest sensitivity of 0.90 among all methodologies. The table provides a clear comparison of the performance of different methodologies on the MSLD dataset. The proposed methodology outperforms the other methodologies in terms of accuracy and F1-measure, while Sahin et al. [36] achieved the highest sensitivity.

## 5. Conclusions and Future Research Directions

This paper presented a methodology for the detection of monkeypox on ordinary skin images. It can be used for the first layer of diagnosis for less privileged areas of the globe. Monkeypox is mostly diagnosed in geographic locations with low health facilities. Therefore, this method is very helpful for such areas. Different deep networks were tested on two publicly available datasets, namely, MSID and MSLD. Domain knowledge transfer was employed using the transfer learning mechanism. The results show encouraging performance of MobileNet and Inception V3. Inception is a deep neural network architecture that is designed to improve the efficiency and accuracy of image classification tasks. In comparison, MobileNet is a lightweight neural network architecture that is designed to run efficiently with limited computational resources.

There are several potential future research directions for AI-based monkeypox detection. Researchers can develop more advanced computer vision algorithms that can accurately detect and classify different stages of monkeypox in images. The dataset used in this study is limited. It can be improved in future research. Researchers can conduct real-world evaluations of AI-based monkeypox detection systems to determine their effectiveness and feasibility in clinical settings. This will require the collection of large datasets and collaboration with healthcare professionals and patients. Moreover, other architectures can also be tested for the mentioned problem.

To improve the accuracy of AI-based monkeypox detection, researchers can incorporate additional data sources, such as patient history, clinical symptoms, and laboratory test results. This can help to provide a more comprehensive picture of the disease and to improve the accuracy of diagnosis.

## Figures and Tables

**Table 1:** Summary of literature review.

Methodology	Disease	Dataset	Results
Alwakid et al. [22]	Melanoma	HAM10000	Accuracy: 0.86 Precision: 0.84 Recall: 0.86 F-score: 0.86
Banasode et al. [23]	Melanoma	ISIC	Accuracy: 0.9690 Sensitivity: 0.9570 Specificity: 0.9020
Yadav et al. [24]	acne	Custom Dataset	Precision: 0.88 Recall: 0.88 F-1 score: 0.88
Sandeep et al. [25]	Chicken Pox, Vitiligo,	Custom Dataset	Accuracy: 0.78 Psoriasis, Acne, Melanoma, Lupus, Ringworm and Herpes
Glock et al. [26]	Measles	Rash Image Dataset	Sensitivity: 0.817 Specificity: 0.97 Accuracy: 0.95
Nafisa et al. [19]	monkeypox	MSLD	Accuracy: 0.79 Precision: 84.00 Recall: 79.00 F1-score: 81.00
Sitaula et al. [30]	Chickenpox, Measles and Monkeypox	Ahsan et al. [31]	Precision: 0.8544 Recall: 0.8547 F1-score: 0.8540 Accuracy: 0.8713
Ariansyah et al. [32]	Measles and Monkeypox	Bala et al. [33]	Accuracy: 0.8333 Precision: 0.84 Recall: 0.8333
Saha et al. [34]	Monkeypox	Ahsan et al. [31]	Accuracy: 0.80
Haque. et al. [35]	Monkeypox	MSLD	Accuracy: 0.83 Precision: 0.90 Recall: 0.89 F1-score: 0.90
Sahin et al. [36]	Monkeypox	MSLD	Accuracy: 91.11 Precision: 0.90 Recall: 0.90 F1-score: 0.90

**Table 2:** Data distribution.

Dataset Name	Monkeypox	Others
MSLD	102	126
MSID	279	198

**Table 3:** Results of different architectures on MSID dataset.

S#	Technique	Accuracy	Loss	Specificity	Sensitivity	Balanced Accuracy
1	Inception V3	0.94	0.234	0.931034	0.952381	0.941708
2	ResNet 50 V2	0.92	0.2307	0.896552	0.952381	0.924466
3	MobileNet V2	0.96	0.1604	0.931034	1	0.965517
4	EfficientNet- B4	0.92	0.2266	0.896552	0.952381	0.924466

**Table 4:** Results of different architectures on MSLD dataset.

S#	Technique	Accuracy	Loss	Specificity	Sensitivity	Balanced Accuracy
1	Inception V3	0.9333	0.246	1	0.88	0.94
2	ResNet 50 V2	0.8889	0.272	0.9	0.88	0.89
3	MobileNet V2	0.8889	0.311	0.9	0.88	0.89
4	EfficientNet- B4	0.8889	0.277	0.9	0.88	0.89

**Table 5:** Comparison of results on the MSLD dataset.

Methodology	Accuracy	Specificity	Sensitivity	F1-Measure
Nafisa et al. [19]	0.79	84.00	79.00	81.00
Haque et al. [35]	0.83	0.90	0.89	0.90
Sahin et al. [36]	0.91	0.90	0.90	0.90
Proposed	0.93	1	0.88	0.94

## Data Availability

Not applicable.
